# LincRNA-p21 suppresses glutamine catabolism and bladder cancer cell growth through inhibiting glutaminase expression

**DOI:** 10.1042/BSR20182372

**Published:** 2019-04-12

**Authors:** Qun Zhou, Hengji Zhan, Fan Lin, Yuhan Liu, Kang Yang, Qunjun Gao, Mengting Ding, Yuchen Liu, Weiren Huang, Zhiming Cai

**Affiliations:** 1 Department of Urology, Shenzhen Second People’s Hospital, Clinical Medicine College of Anhui Medical University, Shenzhen 518035, China; 2 Graduate School, Anhui Medical University, Hefei 230032, China; 3 Department of Urology, The First Affiliated Hospital of Shenzhen University, Shenzhen 518035, China

**Keywords:** bladder cancer, GLS, glutamine catabolism, lincRNA-p21

## Abstract

Long intergenic non-coding RNA p21 (lincRNA-p21) is down-regulated in some solid tumors. Glutamine catabolism plays an important role in cancer development. However, the role of lincRNA-p21 and its association with glutamine catabolism remain unknown in bladder cancer (BC). In the present study, we investigated the involvement of lincRNA-p21 and glutamine catabolism in BC cell growth and found that ectopic linRNA-p21 expression reduced the proliferation and growth of BIU87 and 5637 cells. Opposite results were observed in lincRNA-p21 silenced J82 and T24 cells. The expression of glutaminase (GLS), intracellular level of glutamate and α-Ketoglutarate (α-KG) were negatively regulated by lincRNA-p21. GLS overexpression reversed the suppressive function of lincRNA-p21 on BC cell growth and proliferation. In contrast, GLS reduction by siRNA blunted the viability of lincRNA-p21 lowly expressed BC cells. Furthermore, lincRNA-p21 and GLS abundance dictated the sensitivity of BC cells to bis-2-(5-phenylacetamido-1,2,4-thiadiazol-2-yl)ethyl sulfide (BPTES) treatment. Importantly, reduced lincRNA-p21 expression and increased GLS mRNA level were observed in BC tissues compared with the normal tissues. Our results demonstrate that lincRNA-p21 suppresses the BC cell growth through inhibiting GLS and glutamine catabolism. Targeting this cascade may be a promising treatment strategy for BC patients.

## Introduction

Bladder cancer (BC) is one of the most common malignancies, which represents the ninth of all cancer types throughout the world [1]. It caused more than 160000 deaths in 2012 and the lethality has increased these years [2]. The majority of new cases are diagnosed as non-muscle-invasive BC and the remaining are muscle-invasive BC [3]. The high recurrence rate after standard treatment is the main cause of death, whereas targeted therapies are still lacking for this disease. Therefore, to explore the molecular events contributing to the uncontrolled BC cell growth and proliferation may help us understand the disease progression and develop effective treatment strategies.

Long intergenic non-coding RNA p21 (lincRNA-p21) is initially identified as the direct transcriptional target of P53 [4]. It contains more than 200 nucleotides. Increasing evidences have demonstrated lincRNA-p21 as a tumor suppressor in different cancers, such as prostate cancer and hepatocellular carcinoma [5–8]. However, its involvement in BC is undetermined.

Glutamine is the most abundant amino acid in humans and an important metabolite for cell proliferation and growth [9]. It is first catabolized by glutaminase (GLS) to generate glutamate, followed by glutamate dehydrogenase (GDH) or transaminase-dependent α-ketoglutarate (α-KG) production [10]. α-KG can be then used as the intermediate of tricarboxylic acid (TCA) cycle, which provide the energy, nucleotides, lipids, and amino acid for cell activity [11,12]. Previously, some studies have shown that tumor cells are addicted to glutamine metabolism [11]. Oncogenic Myc activation promotes GLS-dependent glutamine catabolism through transcriptional repression of miR-23a and miR-23b [13]. Mammalian target of rapamycin complex 1 (mTORC1), which is frequently activated in cancers, stimulates the glutamine metabolism in cancer cells [14].

In the present study, we investigated the role of lincRNA-p21 and glutamine catabolism in BC cell growth. The results showed that lincRNA-p21 knockdown promoted BC cell proliferation and growth. GLS expression, glutamate, and α-KG production were enhanced in lincRNA-p21 silenced BC cells. Blockage of GLS with siRNA or bis-2-(5-phenylacetamido-1,2,4-thiadiazol-2-yl)ethyl sulfide (BPTES) suppressed the viability of lincRNA-p21 lowly expressed BC cells. Human BC tissues exhibited reduced lincRNA-p21 and enhanced GLS expression as compared with the normal tissues.

## Materials and methods

Human BC and normal specimens were collected after patients treated with radical cystectomy. All of the patients were diagnosed as transitional cell carcinomas by three independent pathologists. The research has been carried out in accordance with the World Medical Association Declaration of Helsinki. A written informed consent was obtained from each patient and all the experiments were approved by the Ethics Committee of Shenzhen Second People’s Hospital. The relative expression of lincRNA-p21 and GLS was detected by quantitative real-time PCR (qRT-PCR) assay.

### Cell culture

The human BC cells J82, T24, BIU87, and 5637 were obtained from the American Type Culture Collection (Manassas, VA, U.S.A.) and Cell Bank of the Chinese Academy of Sciences (Shanghai, China). All the cells were cultured in Dulbecco’s modified Eagle’s medium (DMEM, Corning, 10-013-CVR, U.S.A.), supplemented with 10% fetal bovine serum (Gibco, 10270-106, U.S.A.), and 1% penicillin and streptomycin solution (Corning, 30-002-CI), in a 37°C incubator with 5% CO_2_.

### Knockdown assay

For lincRNA-p21 knockdown experiments, control shRNA (shCtrl) and shlincRNA-p21, lentivirus particles were designed and purchased from GenePharma company. The target sequences of lincRNA-p21 were GGAGGACACAGGAGAGGCA. siRNA oligos against GLS were purchased from GenePharma company. The BC cells were transfected with 100 nM siGLS or siCtrl using Lipofectamine 2000 (Invitrogen, 11668030, U.S.A.), according to the manufacturer’s instructions. The target sequences of GLS were GCTCTCCTTCGGAGATCTT.

### LincRNA-p21 and GLS overexpression experiments

pSLIK lentivirus system was used for lincRNA-p21 overexpression in BIU87 and 5637 cells. Briefly, human lincRNA-p21 cDNA was subcloned into the pSLIK particles. Virus packaging was performed using 293T cells. Seventy-two hours later, the viruses were harvested and infected the BIU87 and 5637 cells with polybrene. Stable cell lines were seleted by puromycin. GLS was overexpressed in BC cells using pCDH lentivirus vectors. The coding sequence (CDS) of GLS was synthesized and cloned into the pCDH vectors. PSPAX2 and PDM2G served as packaging vectors. These three factors were co-transfected into the 293T cells. Seventy-two hours later, the virus supernatants were collected, filtered, and subjected to infection of BC cells.

### qRT-PCR

Total RNA was extracted from human BC and normal specimens or the cancer cells using TRIzol reagent (Invitrogen, 15596026). A total of 0.5–1 μg of the total RNA was subjected to reverse transcription using M-MLV reverse transcriptase (Promega, M1701, U.S.A.). Subsequent qRT-PCR was performed using TB Green qPCR Mix (Takara, 638319, Japan) on the Bio-Rad machine. The qPCR primer sequences were listed as follows: lincRNA-p21 forward: 5′-CCTGTCCCACTCGCTTTC-3′, and reverse: 5′-GGAACTGGAGACGGAATGTC-3′; GLS forward: 5′-TCTACAGGATTGCGAACGTCT-3′, and reverse: 5′-CTTTGTCTAGCATGACACCATCT-3′; β-actin forward: 5′-GACCTGACTGACTACCTCATGAAG-3′, and reverse: 5′-GTCACACTTCATGATGGAGTTGAAGG-3′. β-actin served as the internal control.

### Western blot assay

The proteins were harvested from indicated cells using TNTE buffer containing protease inhibitor cocktail (Roche, Switzerland). The proteins were boiled, centrifuged, and separated on SDS/PAGE gels and transferred on to PVDF membranes. Then the membranes were blocked by 5% skim milk and incubated with primary antibodies at 4°C overnight. After washing by TBST for three times, the membranes were incubated with the secondary antibodies and the protein abundance was detected by enhanced chemiluminescence (Thermo Fisher Scientific, 34577, U.S.A.). Antibody against GLS was purchased from Abcam (ab93434, United Kingdom). Antibody against β-actin and all the secondary antibodies were obtained from Santa Cruz. Quantitation was analyzed by ImageJ software.

### Cholecystokinin assay

The viability of J82, T24, BIU87, and 5637 cells transfected indicated lentivirus was determined using cholecystokinin (CCK) kit (40203ES80, Yeason, China). In brief, a total of 2000 BC cells containing 200 μl medium was seeded in 96-well plates. Eight hours after seeding (0 h shown in the figure), 20 μl CCK reagent was added into each well and the plates were maintained at 37°C. The optical density (OD) value at 450 nm was measured on a microplate reader. The cell viability of each time was normalized to 0 h.

### BPTES treatment and cell viability analysis

A total of 3000 indicated cells were seeded in 96-well plates and were immediately treated with different concentrations of BPTES (Sigma, 314045-39-1, U.S.A.) (0, 0.5, 2, 5, 10 or 0, 0.1, 0.5, 2, 5 μM). Two days later, culture medium was removed and the 100 μl medium containing 10 μl CCK reagent was added into each well and the plates were maintained at 37°C. Then the OD value at 450 nm was measured on a microplate reader.

### Colony formation assay

Equal number of BIU87 and 5637 cells expressing Ctrl and lincRNA-p21 lentivirus, or 5637 cells expressing Ctrl, lincRNA-p21, and GLS lentivirus were seeded. Ten days after incubating at 37°C, the plates were washed by TBS and fixed with methanol for 30 min. Then the plates were stained with Crystal Violet solution for 20 min. The colonies were photographed using a camera.

### Metabolites measurement

The concentration of glutamate and α-KG were measured by Glutamate Assay Kit (Abcam, ab83389) and α-Ketoglutarate Assay Kit (Sigma, MAK054), respectively. All the experiments were conducted following the manufacturers’ instructions. The intracellular glutamate and α-KG abundance was normalized to the cell number.

### Statistical analysis

All the statistical data were presented as mean ± S.E.M. of at least three independent repeats. The difference between two groups was analyzed by unpaired Student’s *t* test. One-way ANOVA was used for analyzing the difference when more than two groups. Difference was considered significant when *P<0.05; **P<0.01; ***P<0.001.

## Results

### LincRNA-p21 was efficiently overexpressed or knocked down in BC cells

**Figure 1:**
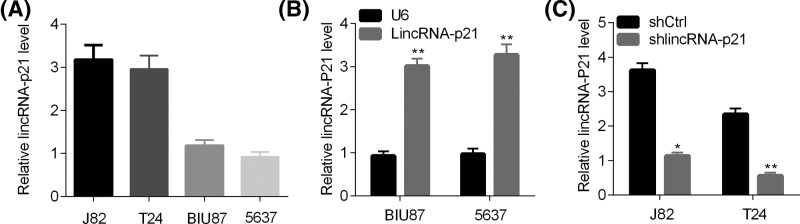
LincRNA-p21 represses the growth and proliferation of BC cells (**A**) qRT-PCR analysis of lincRNA-p21 in J82, T24, BIU87, and 5637 cells. (**B**) qRT-PCR analysis of lincRNA-p21 in control (U6) and lincRNA-p21 overexpressed BIU87 and 5637 cells. **P<0.01 (BIU87 or 5637 cells, lincRNA-p21 vs U6). (**C**) qRT-PCR analysis of lincRNA-p21 in shCtrl and shlincRNA-p21 J82 and T24 cells. *P<0.05 (J82, shlincRNA-p21 vs shCtrl), **P<0.01 (T24, shlincRNA-p21 vs shCtrl).

The function of lincRNA-p21 has been described in other cancers, whereas little is known about its role in BC. First, we analyzed lincRNA-p21 abundance in different BC cells. LincRNA-p21 was highly expressed in J82 and T24 cells comparing with BIU87 and 5637 cells (Figure 1A). Therefore, we overexpressed and silenced lincRNA-p21 in BIU87, 5637 cells and J82 and T24 cells, respectively. qRT-PCR results showed that lincRNA-p21 was efficiently overexpressed or knocked down in BC cells (Figure 1B,C).

### LincRNA-p21 acts as a tumor suppressor in BC cells

**Figure 2:**
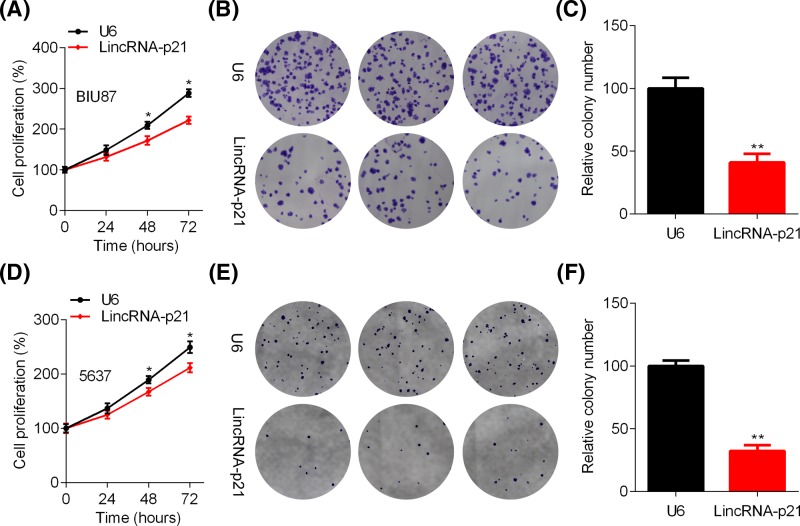
LincRNA-p21 overexpression reduces the proliferation and growth of BIU87 and 5637 cells (**A**–**C**) The viability of control (U6) and lincRNA-p21 overexpressed BIU87 cells was analyzed by CCK (**A**) and colony formation assay (**B**). (**C**) Quantitation of the colony number. *n*=3, **P<0.01. (**D**–**F**) The viability of control (U6) and lincRNA-p21 overexpressed 5637 cells was analyzed by CCK (**D**) and colony formation assay (**E**). (**F**) Quantitation of the colony number. *n*=3, **P<0.01.

**Figure 3:**
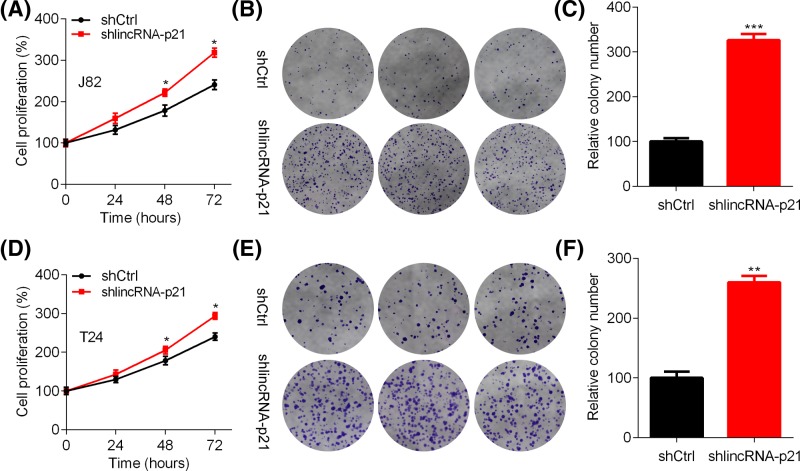
LincRNA-p21 knockdown enhances the proliferation and growth of J82 and T24 cells (**A**–**C**) The viability of shCtrl and shlincRNA-p21 J82 cells was analyzed by CCK (A) and colony formation assay, *P<0.05. (B). (C) Quantitation of the colony number. *n*=3, ***P<0.001. (**D**–**F**) The viability of shCtrl and shlincRNA-p21 T24 cells was analyzed by CCK (**D**) and colony formation assay (**E**). (**F**) Quantitation of the colony number. *n*=3, **P<0.01.

To determine the function of lincRNA-p21 in BC, we subjected control and lincRNA-p21 overexpressed BIU87 and 5637 cells to CCK and colony formation assays. CCK results showed that lincRNA-p21 ectopic expression reduced the viability of BIU87 cells (Figure 2A). In addition, the colony formation capacity of BIU87 cells was blunted by lincRNA-p21 (Figure 2B,C). Consistent results were also observed in control and lincRNA-p21 overexpressed 5637 cells (Figure 2D–F). To verify our results, CCK and colony formation assays were performed in shCtrl and shlincRNA-p21 J82 and T24 cells. The results showed that lincRNA-p21 reduction enhanced the proliferation and colony growth of J82 and T24 cells (Figure 3A–F). Thus, lincRNA-p21 functions as a tumor suppressor in BC cells.

### LincRNA-p21 inhibits GLS expression and glutamine catabolism

**Figure 4:**
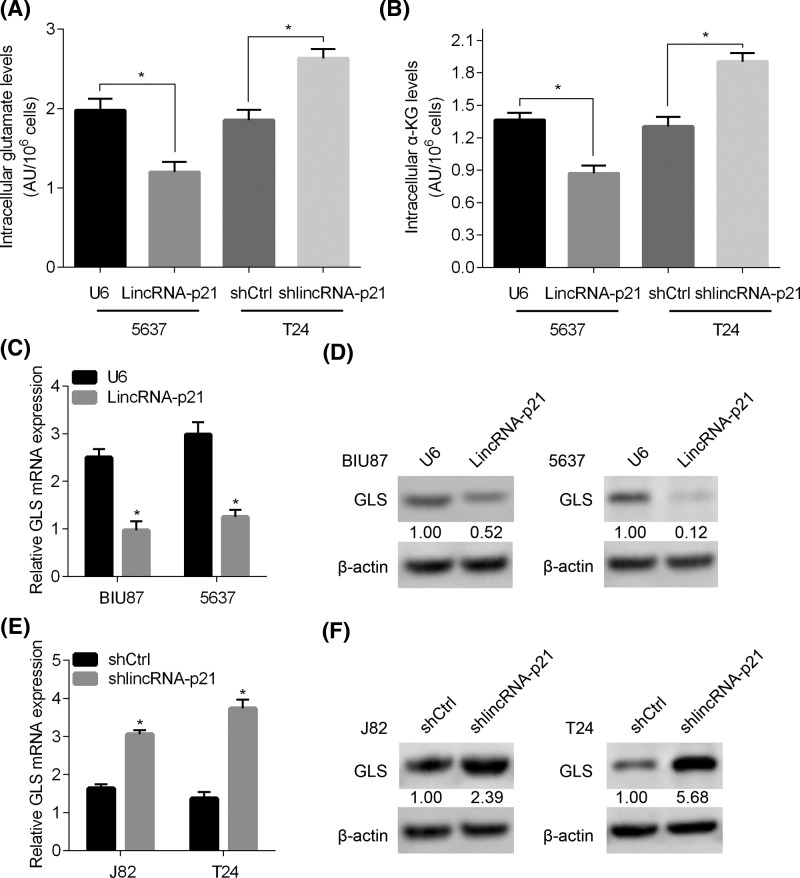
LincRNA-p21 suppresses GLS expression and glutamine catabolism in BC cells (**A**) The intracellular glutamate level was measured in Ctrl and lincRNA-p21 overexpressed 5637 cells and in shCtrl and shlincRNA-p21 treated T24 cells. *P<0.05. (**B**) The intracellular α-KG level was measured in Ctrl and lincRNA-p21 overexpressed 5637 cells and in shCtrl and shlincRNA-p21 T24 cells. *P<0.05. (**C**,**D**) The expression of GLS in Ctrl and lincRNA-p21 overexpressed BIU87 and 5637 cells was determined by qRT-PCR analysis of mRNA level and Western blot analysis of protein level. *P<0.05. (**E**,**F**) The expression of GLS in shCtrl and shlincRNA-p21 J82 and T24 cells was determined by qRT-PCR analysis of mRNA level and Western blot analysis of protein level. *P<0.05.

Glutamine transition to glutamate and α-KG plays an important role in cell proliferation. We initially checked the intracellular glutamate and α-KG level after lincRNA-p21 overexpression and knockdown. We found that intracellular glutamate and α-KG abundance were suppressed by lincRNA-p21 overexpression, while they were stimulated by lincRNA-p21 knockdown (Figure 4A,B). We predicted that the increased glutamate and α-KG level was the result of enhanced GLS. Then the mRNA and protein expression of GLS were determined in these cells. The results showed that GLS was down-regulated in lincRNA-p21 overexpressing BIU87 and 5637 cells (Figure 4C,D). Opposite results were observed in lincRNA-p21 silenced J82 and T24 cells (Figure 4E,F). Taken together, GLS expression and glutamine catabolism were repressed by lincRNA-p21 in BC cells.

### LincRNA-p21 regulation of GLS expression dictates the sensitivity of BC cells to glutamine catabolism inhibition

**Figure 5:**
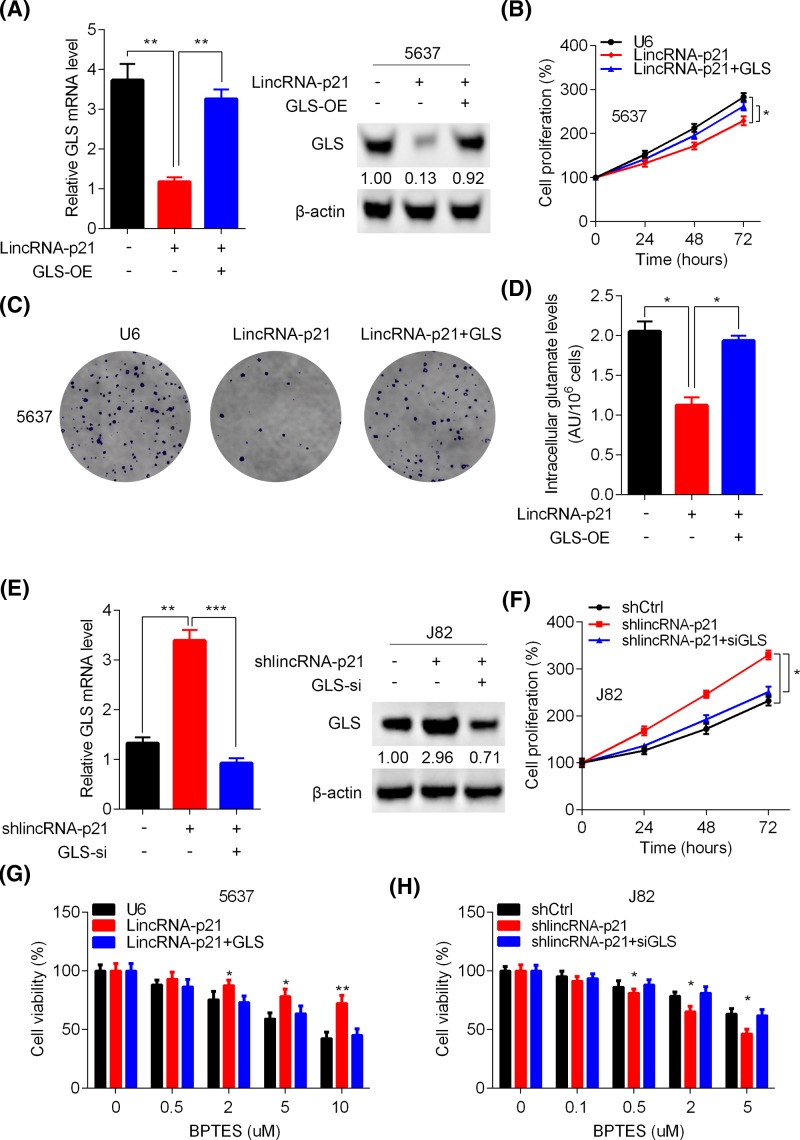
Inhibition of glutamine catabolism reduces the viability of BC cells (**A**) GLS was overexpressed in lincRNA-p21 ectopically expressed 5637 cells and the GLS expression was determined by qRT-PCR and Western blot assay. **P<0.01. (**B**–**D**) The cells described in (**A**) was subjected to CCK assay (**B**), colony formation (**C**) analysis, and intracellular glutamate measurement (**D**). *P<0.05. (**E**) GLS was knocked down in shlincRNA-p21 J82 cells and the GLS expression was determined by qRT-PCR and Western blot assay. **P<0.01, ***P<0.001. (**F**) The cells described in (**E**) were subjected to CCK assay. *P<0.05. (**G**) The cells described in (**A**) were treated with different concentrations of BPTES for 2 days and then were subjected to CCK analysis of cell viability. *P<0.05, **P<0.01. (**H**) The cells described in (**E**) were treated with different concentrations of BPTES for 2 days and then were subjected to CCK analysis of cell viability. *P<0.05, **P<0.01.

To explore whether lincRNA-p21 regulation of GLS and glutamine catabolism participates in BC cell growth and proliferation, we overexpressed GLS in 5637 cells with ectopic lincRNA-p21 expression. GLS was efficiently overexpressed using lentivirus-mediated strategy (Figure 5A). GLS up-regulation rescued the cell growth and proliferation suppressed by lincRNA-p21 (Figure 5B,C). As expected, intracellular glutamate level was enhanced by GLS (Figure 5D). By contrast, we used siRNA to knock down GLS in lincRNA-p21 silenced J82 cells. GLS was efficiently silenced and its down-regulation suppressed the viability of J82 cells. BPTES and its analogs were specific GLS inhibitors [15]. To further verify our results, we used different concentrations of BPTES to treat 5637 and J82 cells for 2 days and the cells were subjected to CCK analysis of cell viability. We found that lincRNA-p21 overexpression reduced the sensitivity of 5637 cells to BPTES treatment, while lincRNA-p21 knockdown enhanced the inhibitory effect of BPTES on J82 cells (Figure 5E,F). In addition, GLS ectopic expression enhanced the effect of BPTES on lincRNA-p21 overexpressed 5637 cells, while GLS knockdown reduced its inhibitory effect on J82 cells (Figure 5G,H). Collectively, lincRNA-p21 regulation of GLS and glutamine catabolism contributes to the BC cell growth.

### Reduced lincRNA-p21 expression and enhanced GLS mRNA level in BC tissues

**Figure 6:**
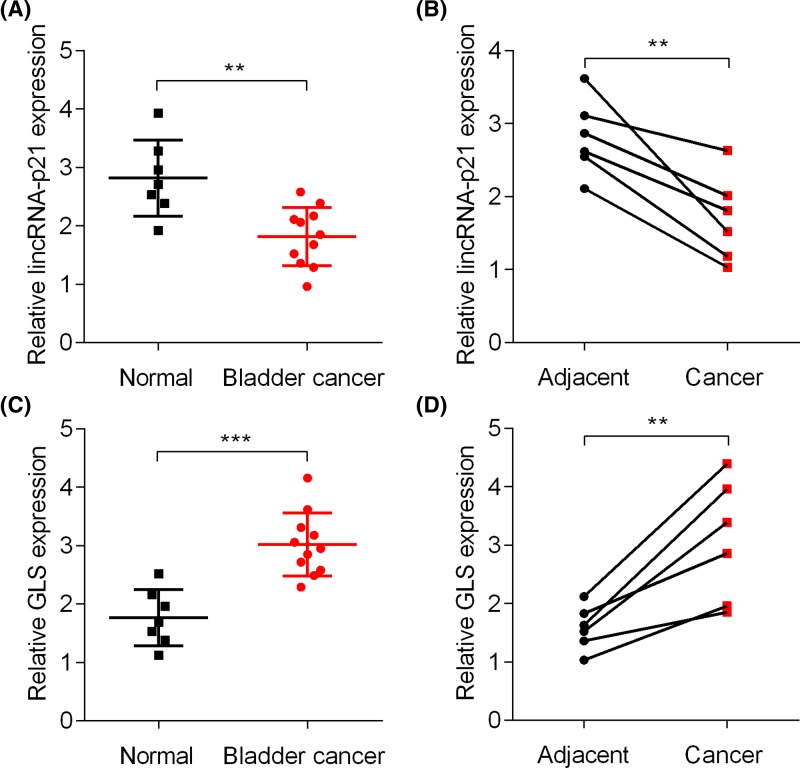
LincRNA-p21 is down-regulated and GLS is up-regulated in BC patients (**A**) qRT-PCR analysis of lincRNA-p21 in BC (*n*=11) and normal tissues (*n*=7). **P<0.01. (**B**) qRT-PCR analysis of lincRNA-p21 in BC and adjacent tissues (*n*=6). **P<0.01. (**C**) qRT-PCR analysis of GLS in BC (*n*=11) and normal tissues (*n*=7). **P<0.01. (**D**) qRT-PCR analysis of GLS in BC and adjacent tissues (*n*=6). ***P<0.001.

To determine the abundance of lincRNA-p21 in BC patients, we collected BC and normal tissues and analyzed the mRNA expression of lincRNA-p21 using qRT-PCR assay. The results showed that lincRNA-p21 was down-regulated in BC tissues compared with normal tissues (Figure 6A). Likewise, lincRNA-p21 expression was lower in BC tissues than that in adjacent tissues (Figure 6B). We then analyzed GLS mRNA expression in these tissues by qRT-PCR assay. GLS was up-regulated in BC the same BC tissues as compared with the normal and their adjacent normal tissues (Figure 6C,D). These results suggest inverse correlation between lincRNA-p21 and GLS expression in BC patients.

## Discussion

The high incidence and lethality makes BC a serious threat to society health. Once diagnosed, the prognosis is gloomy in most of the patients and effective therapies are limited. The development of BC involves multiple changes, including genetic, epigenetic, and metabolism [16]. Therefore, understanding the molecular mechanisms and associated metabolism alterations may help us identify novel targets for this disease. Recently, the role of long non-coding RNAs (lncRNas) has been investigated in BC, such as urothelial carcinoma-associated 1 (UCA1), lINC01296, and MAGI2-AS3 [17–19]. However, the involvement of other lncRNas remains to be identified in BC development.

LncRNAs are regulatory non-coding RNA molecules that play an important role in cell activity. Dysregulation of lncRNAs participates in cancer development. Among them, lincRNA-p21 is a well-characterized one. It was identified as a repressive factor in p53 transcriptional regulation network [4]. Likewise, lincRNA-p21 regulates cell proliferation and apoptosis via enhancing p53 signaling in prostate cancer and atherosclerosis [5,20]. In this study, we investigated the role of lincRNA-p21 in BC and found that silencing of lincRNA-p21 by lentivirus-mediated strategy increased the growth and proliferation of BC cells. Inverse results were observed in lincRNA-p21 overexpressed BC cells. Furthermore, lincRNA-p21 was down-regulated in BC tissues as compared with the adjacent and non-paired normal tissues. These findings suggest that lincRNA-p21 also acts as a tumor suppressor in BC.

Recent studies have shown that lincRNA-p21 regulates signaling transduction in tumor growth. Down-regulated lincRNA-p21 promotes gastric cancer development through activation of YAP independent of Hippo [21]. In hypoxic tumor cells, lincRNA-p21 knockdown represses the autophagy via regulating HIF-1/Akt/mTOR/P70S6K signaling pathway [22]. LincRNA-p21 negatively regulates miR-9/E-cadherin cascade to inhibit the invasion and metastasis of hepatocellular carcinoma [23]. Furthermore, lincRNA-p21 participates in dysregulated glucose metabolism in tumor cells. It binds with VHL and dissociates the HIF-1α from VHL–HIF-1α complex, leading to activation of glycolysis [24]. Here, we reported that glutamine catabolism was suppressed by lincRNA-p21. Intracellular glutamate and α-KG level were decreased in lincRNA-p21 overexpressed BC cells, while they were increased in lincRNA-p21 silenced cells. GLS was down-regulated and up-regulated in lincRNA-p21 overexpressed and silenced BC cells, respectively. These results suggest that lincRNA-p21 regulates glutamine catabolism through GLS. Importantly, the BC tissues which had lowly expressed lincRNA-p21 also showed up-regulated GLS, highlighting the clinical relevance of the lincRNA-p21 regulation of GLS in BC patients. Since GLS-dependent glutamine catabolism was positively regulated by Myc oncogene through its suppression of miR-23a and miR-23b [13], the potential link between lincRNA-p21 and c-Myc/miR-23a/b should be further explored.

Even though GLS expression and glutamine catabolism was mediated by lincRNA-p21, this does not imply that it contributes to the suppressive function of lincRNA-p21 in BC cell proliferation and growth. Thus, we performed rescue experiments. GLS was overexpressed and its ectopic expression reversed the growth and proliferation suppressed by lincRNA-p21. By contrast, GLS knockdown inhibited the enhanced viability of lincRNA-p21 silenced BC cells. The GLS inhibitor BPTES significantly blunted the growth of lincRNA-p21 knockdown, while exhibited minimal effect on lincRNA-p21 overexpressed 5637 cells. Additionally, GLS re-stimulation in the 5637 cells enhanced the inhibitory effect of BPTES and its knockdown reduced the effect. Thus, lincRNA-p21 suppression of GLS and the glutamine catabolism indeed participates in BC cell growth and proliferation.

In summary, we provided for the first time that lincRNA-p21 was involved in glutamine catabolism and BC cell viability. LincRNA-p21 was reduced in BC specimens. Down-regulation of lincRNA-p21 resulted in up-regulated GLS, enhanced glutamine catabolism, and accelerated growth and proliferation of BC cells. Blockage of GLS reversed the viability of lincRNA-p21 silenced BC cells. Thus, lincRNA-p21 is a potential tumor suppressor in BC through regulation of glutamine catabolism which depends on GLS.

## Abbreviations

Akt: Protein kinase B (PKB)
ANOVA: Analysis of Variance
BC: bladder cancer
BPTES: bis-2-(5-phenylacetamido-1,2,4-thiadiazol-2-yl)ethyl sulfide
CCK: cholecystokinin
GLS: glutaminase
HIF-1: hypoxia-inducible factor-1
Hippo: Hippo signaling pathway
lincRNA-p21: long intergenic non-coding RNA p21
LINC01296: long intergenic non-coding RNA 01296
lncRNA: long non-coding RNA
MAGI2-AS3: MAGI2 astisense RNA 3
OD: optical density
P70S6K: Ribosomal protein S6 kinase
qRT-PCR: quantitative real-time PCR
shCtrl: control shRNA
TBST: TRIS BASE SDS Tween-20
VHL: von Hippel-lindau tumor suppressor
YAP: Yes-associated protein
α-KG: α-ketoglutarate
